# Changes in food habits during the transition to retirement: the Whitehall II cohort study

**DOI:** 10.1136/jech-2024-222690

**Published:** 2024-10-01

**Authors:** Hanna Lagström, Mirkka Lahdenperä, Chirsna Ravyse, Tasnime Akbaraly, Mika Kivimaki, Jaana Pentti, Sari Stenholm, Jenny Head

**Affiliations:** 1 University of Turku, Turku, Finland; 2 TYKS Turku University Hospital, Turku, Finland; 3 Centre for Population Health Research, University of Turku, Turku, Finland; 4 Nutrition and Food Research Center, Faculty of Medicine, University of Turku, Turku, Finland; 5 Biology, University of Turku, Turku, Finland; 6 North-West University, Potchefstroom, South Africa; 7 DevPsy, UMS 011 Inserm UVSQ, Paris, France; 8 Department of Epidemiology & Public Health, UCL, London, UK

**Keywords:** DIET, AGING, DEMOGRAPHY, NUTRITION

## Abstract

**Background:**

The transition to retirement is a significant turning point in life, which may lead to changes in food habits.

**Objective:**

To examine changes in red meat, fish, vegetables and fruit consumption during the retirement transition and whether these changes vary between sociodemographic groups.

**Methods:**

The data were from the Whitehall II study, a cohort of 10 308 British civil servants aged 35–55 years at study induction (1985–1988). Data collection has taken place every 2–3 years. Food consumption (n=2484–2491) was assessed with the Food Frequency Questionnaire in the periods before (max. 16 years) and after retirement (max. 16 years). Changes in preretirement and postretirement consumption were compared in the total cohort and subgroups by sex, marital status, preretirement occupation status and financial hardship using linear regression analyses with generalised estimating equations.

**Results:**

Weekly red meat consumption was stable before retirement but increased after retirement (p=0.02), especially among women, single and lower occupational status participants. Fish consumption increased during the follow-up and the increase was steeper before retirement than postretirement period (p=0.02). Vegetable and fruit consumption also increased during the entire follow-up, but more strongly during preretirement than postretirement period (p<0.001 for both).

**Conclusion:**

The transition to retirement is accompanied by favourable (increase in fruit, vegetable and fish) and unfavourable (increase in red meat) dietary changes, varied to some extent by sex, marital status and preretirement occupational status. Our findings suggest that attention should be paid to this transitional phase to promote eating habits in accordance with the recommendations for retirement.

**WHAT IS ALREADY KNOWN ON THIS TOPIC**

- Transition to retirement is a significant turning point in life, which is accompanied by changes in many aspects of life. These changes may also predispose or lead to changes in food habits.

**WHAT THIS STUDY ADDS**

- The transition to retirement is accompanied by favourable (increase in fruit, vegetable and fish consumption), but also unfavourable (increase in red meat consumption) dietary changes, and these changes varied to some extent by sex, marital status and preretirement occupational status.

**HOW THIS STUDY MIGHT AFFECT RESEARCH, PRACTICE OR POLICY**

- This study highlights the need for strategies to promote eating habits in accordance with the recommendations for retirement.

## Introduction

 Transition to retirement is a significant turning point in life, which is accompanied by changes in many aspects of life, including time availability, daily routines, income streams and social networks.^1^ These changes may also predispose or lead to changes in food habits. However, research in this area remains scarce, and the results have been inconsistent.^2 3^

Some prospective studies have found worsening diets, such as a decrease in fruit and vegetable consumption^4 5^ or a reduction in adherence to nutritional guidelines^6^ after retirement. In a recent Swiss study, retirement was associated with an unhealthier overall dietary intake measured with a Mediterranean Diet Score.^7^ Other studies have reported improvements in food habits after retirement, such as better adherence to recommendations,^8^ an increase in consumption of vegetables^9^ or an increase in fish intake^7 10^ or no changes in food habits.^11^

Heterogeneity in results could be explained by differences in the length of follow-up, which have varied from a few years^5^,^811^ to up to 20 years.^4 9^ The sample size has also varied from a few hundred to thousands^4 6 8 9 11^ and only a few studies have had multiple follow-up measurements.^4 9^ Moreover, most of the studies on food habits and retirement have focused only on fruit and vegetable or fish consumption, but not the consumption of red meat^4 5 9^ or only specific nutrients, such as fat, protein or fibre.^6 7^ Since much attention in the current dietary recommendations is paid to decreasing red meat consumption and increasing fish, vegetable and fruit consumption,^12 13^ it would be important to examine how consumption of these specific food groups changes during retirement.

A further source of heterogeneous findings may stem from the different sociodemographic composition of the study samples. This is important because dietary preferences and food choices may vary across different sociodemographic factors. Retirement may have different impacts on diet related to financial and health reasons.^614^,^16^ Studies suggest that men and individuals with lower education tend to consume more red meat than women and those with higher education.^17^,^19^ Fish consumption is more common among women and those with higher incomes and higher socioeconomic positions.^17 18 20 21^ In addition, vegetable and fruit consumption is higher among women and those with higher education, living in a relationship and higher income.^17 22 23^ However, little is known about how these sociodemographic factors affect changes in food habits during the transition to retirement.^6 8 14^

In the present study, we aimed to examine changes in fish, red meat, vegetables and fruit consumption as indicators of recommended food habits before and after retirement transition over a 32-year follow-up. In addition, we examined whether these changes vary by sex, preretirement occupational position, marital status and financial hardship.

## Methods

### Study population

The data were from the Whitehall II study e,^24^ an ongoing prospective cohort of 10 308 (6895 men and 3413 women) British civil servants aged 35–55 years at study induction (phase 1: 1985–1988). This study uses data from phases 3 (1991–1993, n=8352), 5 (1997–1999, n=5391), 7 (2003–2004, n=5663) and 9 (2007–2009, n=5396), when comparable dietary assessments were conducted.

### Retirement

The participants reported their employment status at each phase. Participants were considered to be employed if they were either still in civil service or paid employment elsewhere, either full or part time. Participants were considered retired if they moved from employment to retirement directly or from employment to unemployed/other and then to retirement. We excluded those participants who retired due to health reasons. Those participants who retired directly from civil service provided the exact year of exit, that is, retirement. For those who retired from employment other than civil service, the exact year of retirement was not known, but a midpoint between the last study phase still working and the subsequent study phase no longer working was used as the year of retirement. We centred the data around the year of retirement to examine changes in food habits before and after retirement.

Only participants retiring, who completed at least one dietary assessment before and at least one assessment after retirement and who had information on covariates, were selected for the analyses. The final sample size included 2484–2491 participants depending on the food group, with a maximum of −16 to +16 years from retirement. Participants provided data on dietary measures at 3.7 (SD 0.6) times of the possible four study waves during a 32-year follow-up. A flow chart summarising inclusion and exclusion criteria for the present study samples is presented in online supplemental figure 1.

### Consumption of red meat, fish, vegetables and fruits

A machine-readable Food Frequency Questionnaire (FFQ) assessed participants’ food consumption based on one used in the US Nurses’ Health study.^25^ Response to all items was on a 9-point scale, ranging from ‘never or less than once per month’ to ‘six or more times per day’. The selected frequency category for each food item was converted to a weekly intake by using the following frequencies: 0 (never or <once/month), 0.5 (1–3/month), 1 (once a week), 3 (2–4/week), 5.5 (5–6/week), 7 (once a day) and 14 (2–3/day or 4–5/day or /6+/day). For example, a participant who reported consumption of a food item 2–3 times per day or more was given a weekly intake of 14 units/portions.

We selected four food groups: red meat, fish, vegetables and fruits because their optimal consumption has been raised as crucial dietary challenges for healthy ageing in Western populations (online supplemental table 1). The weekly consumption frequency (0–14) of all selected food items was summed together to get each food group’s total consumption frequency (fish/red meat/vegetables/fruits). To be included in the summary dietary group variable, the participant had to give a response to at least half of the selected food items in each food group.

### Sociodemographic factors

Information about all sociodemographics and socioeconomic indicators, that is, sex, retirement age, occupational status, marital status (married/cohabiting or not) and financial hardship (yes/no) was obtained from a general questionnaire. Information from the study wave immediately before retirement was used to define marital status and financial hardship.

To index the occupational status (administrative (highest)/professional/executive/clerical/support (lowest)), the participant’s civil service employment grade was used. Information from occupational status was primarily gained from a study wave just before retirement, but if it was missing information was supplemented from a previous study wave. Marital status was dichotomised as married/cohabiting versus single (including never married, separated, divorced or widowed). Financial hardship was based on a question covering the frequency of not having enough money to afford adequate food or clothing (five responses, ‘never’ to ‘always’). Responses ‘always’, ‘often’ and ‘sometimes’ were combined to group with financial hardship (yes) and ‘seldom’ and ‘never’ to group with no financial hardship (no).

### Statistical methods

Characteristics of the study participants at the last measurement before retirement are presented as proportions for sociodemographic variables and means with 95% CI for weekly consumption rates of four food groups.

Changes in fish, red meat, vegetables and fruit consumption before and after retirement were assessed using linear regression analyses with generalised estimating equations (GEEs). As repeated measurements are used, the GEE model controls for the intraindividual correlation between repeated measurements.^26^ The model uses an exchangeable correlation structure and is not sensitive to measurements missing completely at random (MCAR). The MCAR assumption was tested by examining the probability that a participant will drop out is related to his/her previous food consumption (fish, fruits, vegetables and red meat separately). We observed that consumption of certain food in the previous study wave did not predict participation in the following study wave (p for all food items >0.05) supporting the MCAR assumption in the current data.

Due to non-normal distributions, natural logarithmic transformation was performed for all food group variables. To study changes in each food group’s consumption before and after retirement, we constructed two consecutive periods: the preretirement period (from −16 to <0 years to retirement) and the postretirement period (from 0 to +16 years from retirement). The periods were non-overlapping to allow testing whether the changes (slopes) in each food group consumption differed between the preretirement period and postretirement periods. The statistical significance of these changes was tested using a period×time interaction term, where time measured as years before and after retirement was treated as a continuous variable.

We further investigated if the changes in each food group consumption differed between preretirement and postretirement periods by sex, occupational status, marital status and financial hardship. This was done by conducting separate analyses for each food group including three-way interaction, sociodemographic term×period×time in the model. The adjusted slopes and their 95% CIs were calculated to represent the changes in fish, red meat, vegetables or fruit consumption during preretirement and postretirement periods (in logarithmic scale). In addition to variables time and period in the models (and two-way and three-way interactions), all analyses were adjusted for retirement age, occupation, sex and retirement year (1990–1994/1995–1999/2000–2004/2005–2009). The figures show predicted means and 95% CIs and are shown in the original scale. All analyses were conducted by using the SAS V.9.4 Statistical Package (SAS Institute).

## Results

The mean age of participants just before retirement was 57.1 years (SD 4.4). The majority of this population were men (71%), had a high occupational status (87%), were married or cohabiting (78%), had no financial hardship (92%) and were white (93%) from their ethnic background (online supplemental table 2). Before retirement, women ate fish, vegetables and fruit more and red meat less frequently than men. Participants with the highest occupational status ate fish, vegetables, and fruit more and red meat less frequently than those with the lowest status. Married or cohabited people consumed red meat and vegetables more frequently than single participants (online supplemental table 2).

### Red meat consumption and retirement

**Figure 1:**
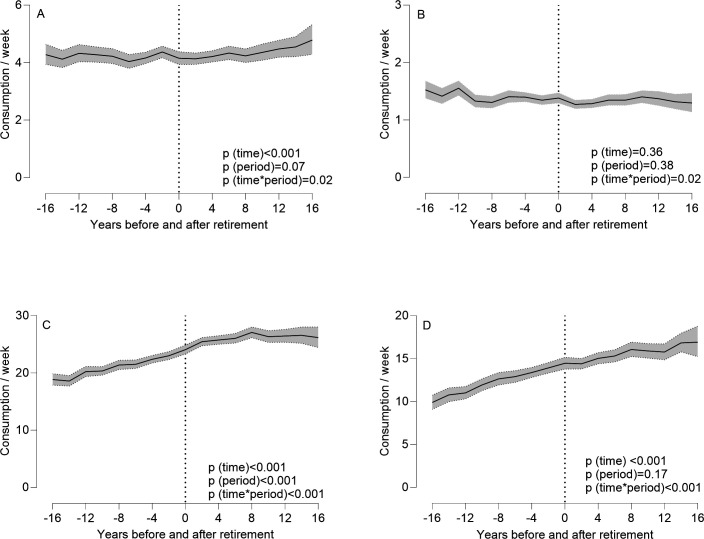
Changes in weekly consumption frequency of red meat (**A**), fish (**B**), vegetables (**C**) and fruits (**D**) during retirement periods (−16 to +16 years from retirement). Models were adjusted for sex, age and occupational status prior to retirement and retirement year.

Weekly red meat consumption frequencies differed between preretirement and postretirement periods during the 32-year follow-up, staying stable before retirement but increasing significantly after retirement (interaction period×time, p=0.02, table 1, figure 1A). There were differences by sex and marital status (table 2, online supplemental figure 2A,C). The increase in red meat consumption during the postretirement period was higher in women than men (interaction: sex×period×time, p=0.01) and in single than married participants compared with the preretirement period (interaction: marital status×period×time, p=0.04). In addition, those in clerical and support occupations showed increased red meat consumption, which was not observed in the higher occupational groups (online supplemental figure 2B). Still, the interaction occupational status×period×time was only borderline significant, p=0.06 (table 2). No difference in this trend by financial hardship was observed (interactions financial hardship×period×time p=0.40, table 2, online supplemental figure 2D). Overall, men and married participants consumed red meat more often than women and single participants (p<0.001 for both, online supplemental figure 2A,C).

**Table 1:** Mean level and changes in weekly consumption of four food groups during the retirement periods

Term→	Preretirement period	Postretirement period	Preretirement period	Postretirement period	P value
Food group↓	Mean (95% CI)	Mean (95% CI)	Slope/year (95% CI)	Slope/year (95% CI)	Period×time
Red meatn=2484	4.22 (4.04, 4.41)	4.08 (3.91, 4.26)	0.0001 (−0.0030, 0.0033)	0.0052 (0.0021, 0.0083)	0.02
Fishn=2486	1.80 (1.71, 1.88)	1.79 (1.72, 1.86)	0.0121 (0.0093, 0.0148)	0.0064 (0.0036, 0.0092)	0.003
Vegetablesn=2491	23.55 (22.92, 24.19)	24.78 (24.18, 25.39)	0.0148 (0.0124, 0.0171)	0.0066 (0.0044, 0.0089)	<0.001
Fruitsn=2488	14.66 (14.10, 15.25)	14.36 (13.84, 14.90)	0.0213 (0.0178, 0.0248)	0.0103 (0.0070, 0.0136)	<0.001

Notes: Slopes (change per year) during preretirement- and post-retirement periods are in logarithmic scale (response variables log-transformed) but means are back-transformed and report the weekly consumption rate of each dietary component. Analyses were adjusted for sex, retirement age, occupational status and retirement year.

**Table 2:** Mean level and changes in weekly consumption of four food groups during the retirement periods by sex, occupational status, marital status and financial situation

	Preretirement period	Postretirement period	Preretirement period	Postretirement period	P value
	Mean (95% CL)	Mean (95% CL)	Slope/year (95% CL)	Slope/year (95% CL)	Term×period×time
Red meat					
Sex					0.01
Men	4.97 (4.71, 5.23)	4.86 (4.62, 5.12)	0.0003 (−0.0031, 0.0038)	0.0012 (−0.0022, 0.0047)	
Women	3.52 (3.25, 3.81)	3.31 (3.07, 3.57)	−0.0004 (−0.0075, 0.0068)	0.0138 (0.0077, 0.0198)	
Occupational status					0.06
Administrative	4.16 (3.92, 4.42)	4.09 (3.85, 4.34)	0.0026 (−0.0014, 0.0066)	0.0020 (−0.0021, 0.0061)	
Professional	3.89 (3.65, 4.14)	3.89 (3.66, 4.11)	−0.0051 (−0.0103, 0.0000)	0.0044 (−0.0006, 0.0094)	
Clerical/support	4.83 (4.27, 5.47)	4.01 (3.57, 4.49)	0.0087 (−0.0034, 0.0209)	0.0182 (0.0092, 0.0271)	
Marital status					0.04
Married/cohabiting	4.39 (4.18, 4.60)	4.28 (4.09, 4.48)	0.0013 (−0.0020, 0.0047)	0.0035 (0.0001, 0.0069)	
Single	3.82 (3.47, 4.19)	3.59 (3.30, 3.90)	−0.0036 (−0.0119, 0.0047)	0.0108 (0.0038, 0.0178)	
Financial hardship					0.40
Yes	4.20 (3.57, 4.92)	3.68 (3.16, 4.25)	0.0003 (−0.0141, 0.0148)	0.0127 (0.0003, 0.0252)	
No	4.23 (4.04, 4.42)	4.13 (3.95, 4.31)	0.0001 (−0.0032, 0.0033)	0.0046 (0.0014, 0.0078)	
Fish					
Sex					0.09
Men	1.57 (1.48, 1.66)	1.53 (1.45, 1.62)	0.0139 (0.0108, 0.0171)	0.0063 (0.0031, 0.0095)	
Women	2.00 (1.85, 2.15)	2.07 (1.93, 2.22)	0.0066 (0.0010, 0.0122)	0.0065 (0.0011, 0.0119)	
Occupational status					0.61
Administrative	2.16 (2.04, 2.29)	2.15 (2.03, 2.27)	0.0120 (0.0082, 0.0158)	0.0052 (0.0012, 0.0092)	
Professional	1.72 (1.61, 1.84)	1.72 (1.61, 1.82)	0.0133 (0.0090, 0.0177)	0.0076 (0.0034, 0.0117)	
Clerical/support	1.50 (1.31, 1.72)	1.53 (1.35, 1.71)	0.0068 (−0.0023, 0.0158)	0.0066 (−0.0018, 0.0150)	
Marital status					0.82
Married/cohabiting	1.77 (1.68, 1.86)	1.75 (1.66, 1.83)	0.0120 (0.0089, 0.0150)	0.0064 (0.0033, 0.0095)	
Single	1.86 (1.69, 2.03)	1.91 (1.76, 2.07)	0.0130 (0.0065, 0.0195)	0.0063 (0.0002, 0.0124)	
Financial hardship					0.28
Yes	1.85 (1.59, 2.14)	1.79 (1.55, 2.06)	0.0063 (−0.0037, 0.0162)	0.0083 (−0.0028, 0.0195)	
No	1.78 (1.70, 1.87)	1.78 (1.71, 1.86)	0.0123 (0.0094, 0.0152)	0.0062 (0.0033, 0.0091)	
Vegetables					
Sex					0.73
Men	22.19 (21.46, 22.95)	23.24 (22.51, 23.99)	0.0153 (0.0126, 0.0180)	0.0068 (0.0042, 0.0095)	
Women	24.84 (23.73, 26.00)	26.47 (25.47, 27.51)	0.0134 (0.0088, 0.0180)	0.0062 (0.0021, 0.0102)	
Occupational status					0.38
Administrative	24.82 (23.93, 25.74)	26.44 (25.57, 27.34)	0.0146 (0.0114, 0.0179)	0.0055 (0.0024, 0.0087)	
Professional	23.83 (22.93, 24.75)	24.87 (24.02, 25.76)	0.0160 (0.0124, 0.0196)	0.0070 (0.0036, 0.0103)	
Clerical/support	21.83 (20.20, 23.58)	22.70 (21.28, 24.21)	0.0097 (0.0011, 0.0184)	0.0091 (0.0014, 0.0169)	
Marital status					0.11
Married/cohabiting	24.88 (24.14, 25.64)	25.87 (25.16, 26.60)	0.0160 (0.0134, 0.0187)	0.0065 (0.0039, 0.0090)	
Single	20.16 (19.04, 21.32)	22.21 (21.14, 23.32)	0.0102 (0.0052, 0.0151)	0.0069 (0.0021, 0.0117)	
Financial hardship					0.54
Yes	23.36 (21.16, 25.77)	25.16 (23.12, 27.37)	0.0093 (0.0002, 0.0184)	0.0049 (−0.0048, 0.0146)	
No	23.55 (22.91, 24.21)	24.77 (24.15, 25.40)	0.0152 (0.0127, 0.0176)	0.0067 (0.0044, 0.0090)	
Fruits					
Sex					0.70
Men	12.73 (12.10, 13.39)	12.20 (11.60, 12.83)	0.0238 (0.0198, 0.0278)	0.0141 (0.0102, 0.0180)	
Women	16.66 (15.63, 17.75)	17.17 (16.23, 18.16)	0.0138 (0.0069, 0.0207)	0.0021 (−0.0036, 0.0078)	
Occupational status					0.08
Administrative	16.18 (15.37, 17.03)	15.84 (15.07, 16.65)	0.0250 (0.0206, 0.0294)	0.0092 (0.0046, 0.0139)	
Professional	14.23 (13.44, 15.07)	13.75 (13.02, 14.52)	0.0193 (0.0134, 0.0253)	0.0148 (0.0097, 0.0199)	
Clerical/support	13.35 (11.94, 14.91)	13.95 (12.71, 15.29)	0.0099 (−0.0021, 0.0218)	−0.0009 (−0.0097, 0.0080)	
Marital status					0.99
Married/cohabiting	14.67 (14.02, 15.34)	14.13 (13.53, 14.75)	0.0231 (0.0191, 0.0270)	0.0127 (0.0090, 0.0163)	
Single	14.46 (13.37, 15.61)	14.84 (13.82, 15.92)	0.0141 (0.0064, 0.0218)	0.0037 (−0.0033, 0.0107)	
Financial hardship					0.09
Yes	13.99 (12.03, 16.24)	13.52 (11.95, 15.28)	0.0150 (−0.0009, 0.0309)	0.0204 (0.0092, 0.0315)	
No	14.73 (14.16, 15.32)	14.41 (13.87, 14.97)	0.0218 (0.0182, 0.0254)	0.0096 (0.0062, 0.0130)	

Notes: Slopes during preretirement- and post-retirement periods are in logarithmic scale (response variables log-transformed) but means are back-transformed and report the weekly consumption rate of each dietary component. Analyses were adjusted for sex, retirement age, occupational status and retirement year.

### Fish consumption and retirement

During the whole 32-year follow-up, weekly frequencies of fish consumption increased. The increase was stronger before retirement than after retirement (interaction period×time, p=0.003, table 1, figure 1B). No differences in fish consumption trends were observed by sex, occupational status, marital status and financial hardships (interactions sociodemographic term×period×time, p>0.05, table 2, online supplemental figure 3A–D). However, women and participants from higher occupational status consumed fish more frequently during the entire follow-up than men and participants from lower occupational status (p<0.001 for both, online supplemental figure 3A,B).

### Vegetable consumption and retirement

During the entire follow-up, weekly vegetable consumption increased, but more strongly during preretirement than postretirement period (interaction period×time, p<0.001, table 1, figure 1C). No differences were observed in this trend by sex, occupational status, marital status or financial hardship (interaction sociodemographic term×period×time, p>0.05, table 2, online supplemental figure 4A–D). In general, women, participants from higher occupational status and married participants consumed vegetables more frequently than men, participants from lower occupational status and single participants (p<0.001 for all, online supplemental figure 4A–D).

### Fruit consumption and retirement

Weekly fruit consumption frequencies increased throughout the follow-up period, and more strongly during the preretirement than postretirement periods (interaction period×time, p<0.001, table 1, figure 1D). There were no differences in this trend by sex, occupational status, marital status or financial hardship (interactions sociodemographic term×period×time, p>0.05, table 2, online supplemental figure 4A–D). Overall, women and participants from higher occupational status consumed more frequently fruits than men and participants from lower occupational status (p<0.001 for both, online supplemental figure 5A,B).

## Discussion

In the present study consumption of red meat increased after retirement, especially among women, those with lower occupational status and married participants. Fish consumption increased during the whole follow-up and more strongly before than after retirement. Vegetable and fruit consumption also increased throughout the follow-up period, but the increase was slightly greater during the preretirement period. There were no differences in fish, vegetable and fruit consumption patterns across sociodemographic factors.

The novelty value of this study is an extended 32-year follow-up, which enabled us to track long-term changes before and after retirement in food groups which are emphasised in the current dietary recommendations. Furthermore, few studies have investigated how sociodemographic variables are associated with food habits during the retirement transition. Our findings confirm previous observations suggesting that meat and processed meat consumption tend to be more common among men and those with lower education than in women or individuals with higher education.^17^,^19^ In our study, the increase in red meat consumption postretirement period was higher in women than men and in single than married participants, as reported earlier.^27^ The red meat consumption appears to stay relatively stable across the whole study period, even though a decrease in red meat consumption was observed during the bovine spongiform encephalopathy crisis in the 1990s.^28^ We additionally found increased red meat consumption after retirement among the clerical and support occupational groups but not among those with higher-grade occupations, which might be associated with better health awareness among the higher socioeconomic groups. Interestingly, our 32-year follow-up, which ended in 2009, did not capture any reduction in red meat consumption. This might be because the strong recommendations to reduce meat consumption are relatively recent and have been emphasised from health and environmental perspectives.

In the UK, consumption of total ﬁsh and ﬁsh products has remained relatively stable over the past few decades.^29^ We found that the fish consumption increased steadily during the whole follow-up, and more strongly before than after the retirement as has been also in a Swiss study.^7^ A recent systematic review concluded that seafood consumers are more likely to be older, have higher socioeconomic status or have higher education than non-seafood consumers.^21^ In our study, participants from the higher occupational status group consumed fish more frequently throughout the follow-up than participants from the lower occupational status as has been observed earlier.^17 18 20 21 30^ In our study, women consumed fish more frequently during the follow-up than men, a finding which persisted over the entire follow-up and has also been reported for shorter follow-ups^20^ but is in contrast to findings reported from Finland.^4^ In our earlier study from Whitehall II,^20^ fish consumption among women was greater than in men, and the differences stayed similar throughout the follow-up. This may indicate healthier eating patterns among women than men in general.^31 32^ Contrary to our findings, higher fish consumption among married participants has been found.^33^ Lower incomes may limit the purchase of relatively expensive foods such as ﬁsh, but this was not supported by this study.

Some years ago (2016–2019) adults aged 19–64 years in UK consumed fruit and vegetables on average 4.3 portions per day, which appears to be slightly less than at the beginning of our study.^34^ We observed an increasing trend in fruit and vegetable consumption during the entire 32-year follow-up. Interestingly, we found the increase was greater in the preretirement than postretirement period, as reported earlier for vegetables.^9^ On the other hand, some previous prospective studies have found a decrease in (fruit) and vegetable consumption during the transition to retirement.^4^,^635^ Furthermore, an Australian study found no changes in fruit consumption on retirement.^11^ We found no sociodemographic differences in the pattern of changes in vegetable and fruit consumption. This is in contrast with a study from Finland that observed that vegetable intake decreased in women and fruit consumption increased among men after retirement.^4^ However, female sex, higher education, living in a relationship and higher income have been associated with greater vegetable and fruit consumption in some previous studies^17 22 23^ as well as observed in our study. Living with others has also been associated with healthier dietary behaviour compared with living alone.^22 36 37^ It is possible that the transition to retirement may have different impacts on diet related to financial and health reasons, such as retiring with a reasonable pension compensation compared with retiring with financial difficulties or poor health status.

The major strengths of this study are its large sample size, prospective design and repeated measurements of diet over 32 years, which allowed us to examine changes during a very long period around the retirement transition. Reporting red meat consumption (eg, beef, lamb, pork, processed meat products) as a separate food group is a further strength because this food group is highlighted in many current dietary recommendations and those on the sustainability of eating patterns. Many previous studies have used diet quality scores^6 7 38^ to describe dietary habits, but food groups are a good illustration of dietary patterns.^39^ In addition, the strength of using food groups is that they are not tied to any nutrition calculation programmes or food databases.

This study also has several limitations. First, the analysis was conducted in an urban British population, the majority of the participants being male and white-collar workers, limiting the generalisability of the findings. In addition, statistical power to detect differences between sexes may be limited due to the low number of female participants, as it was already in the original data. The FFQ provided information on the usual consumption frequency of food items. Indeed, the FFQ remained more or less similar at all follow-up survey phases, and the short FFQ has been considered a suitable instrument for monitoring changes in food patterns at a group level and for frequently consumed foods in particular.^25^ Although FFQ is open to measurement errors common to all self-reported dietary assessments, it remains a mainstream method of analytic epidemiological studies.^40^ As the participants retired several years ago, the findings do not necessarily reflect the eating habits of today’s 60-year-old UK population. Consequently, more up-to-date research is needed because eating habits change over time, and the effect of retirement may vary according to the country of the study.

## Conclusions

Our study showed that the transition to retirement is accompanied by favourable and unfavourable dietary changes throughout the follow-up. Indeed, the dietary pattern of changes was partly dependent on sociodemographic factors. Identifying transitional periods in the life course, like retirement, could improve the implementation of dietary interventions. For example, interventions before the age of retirement focused to optimise dietary patterns with reduced budget could be feasible. However, more up-to-date research is needed because eating habits change over time, and the effect of retirement may vary according to the country of the study.

## supplementary material

10.1136/jech-2024-222690 online supplemental file 1

## Data Availability

Data are available on reasonable request.
