# Supplementary material for: Changes in food habits during the transition to retirement: the Whitehall II cohort study
Source: J Epidemiol Community Health. 2024 Oct 1;79(2):e222690. doi: 10.1136/jech-2024-222690 (PMC11874461; doi:10.1136/jech-2024-222690)

Changes in food habits during the transition to retirement: the Whitehall II cohort study

Lagström H. et al.

Online Supplementary Material

Supplemental Figure 1. Flowchart summarizing exclusion and inclusion criteria for present study samples from the Whitehall II cohort study.

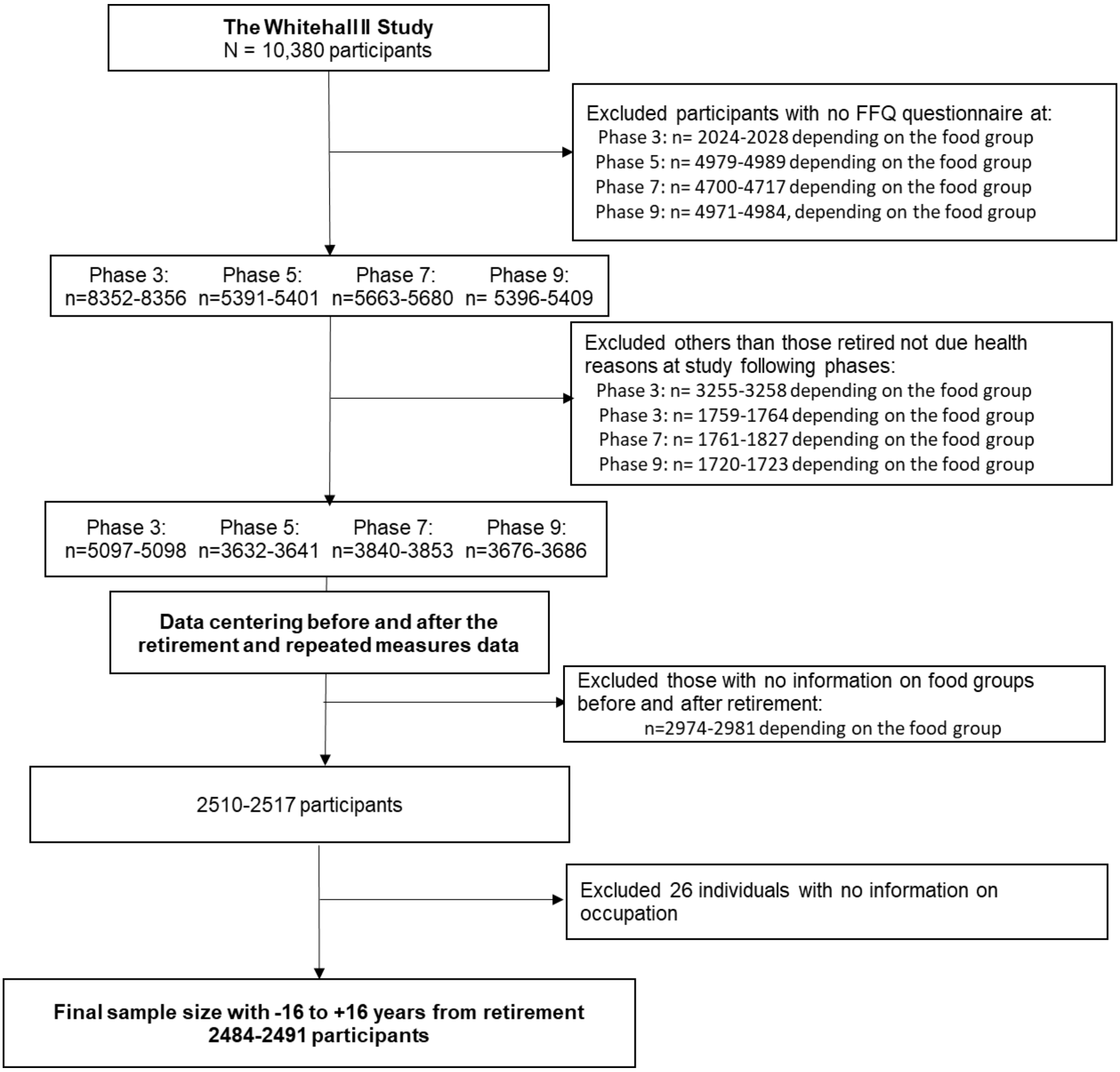

Supplementary table 1. Selected food items by the fish, red meat, vegetables and fruits food groups.

	**Fish**	**Red / processed meat**	**Vegetables**	**Fruit**
Included	Oily fish (fresh/ canned)<br>Shellfish<br>White fish (fresh / frozen)	Bacon<br>Beef (roast/ steak)<br>Beef burgers<br>Corned beef<br>Ham<br>Lamb (roast/ chops/ stew)<br>Liver (also pate)<br>Pork (roast/chops/ stew)<br>Sausages	Brussels sprouts<br>Baked beans<br>Beans (green / broad)<br>Broccoli<br>Cabbage<br>Carrots<br>Cauliflower<br>Dried lentils /beans<br>Green salad<br>Leeks<br>Marrows<br>Courgettes<br>Mushrooms<br>Onions<br>Parsnip/ turnip/swedes<br>Peas<br>Spinach<br>Spring greens/ kale<br>Sweet peppers<br>Tomatoes	Apples<br>Bananas<br>Grapefruit<br>Grapes<br>Melon<br>Oranges<br>Satsumas<br>Peaches<br>Plums<br>Apricots<br>Pears<br>Strawberries<br>Raspberries
Excluded	Fried fish<br>Fish fingers<br>Fish cakes	Meat soup<br>Savory pies	Tofu<br>Coleslaw<br>Soymilk<br>Garlic<br>Nuts	

Supplementary table 2. Pre-retirement weekly consumption rates of fish, red meat, vegetables and fruits by sex, occupational status, marital status and financial hardship.

		Red meat (n=2484)		Fish (n=2486)		Vegetables (n=2491)		Fruits (n=2488)	
**Characteristics**	% (n)	Mean (95% CL)	P	Mean (95% CL)	P	Mean (95% CL)	P	Mean (95% CL)	P
Sex									
Men	70.6 (1759)	4.97 (4.73, 5.22)	<0.001	1.48 (1.40, 1.57)	<0.001	21.04 (20.39, 21.71)	<0.001	11.72 (11.17, 12.31)	<0.001
Women	29.4 (732)	3.53 (3.32, 3.75)		1.96 (1.84, 2.08)		24.02 (23.15, 24.92)		16.21 (15.32, 17.14)	
Occupational status									
Administrative	45.6 (1135)	4.12 (3.89, 4.36)	0.04	2.05 (1.94, 2.17)	<0.001	23.85 (23.03, 24.70)	0.001	15.09 (14.30, 15.92)	<0.001
Professional	41.7 (1039)	3.95 (3.76, 4.16)		1.63 (1.54, 1.72)		22.79 (22.09, 23.52)		13.34 (12.71, 14.00)	
Clerical	12.7 (317)	4.54 (4.14, 4.97)		1.48 (1.33, 1.63)		20.90 (19.73, 22.13)		13.04 (11.93, 14.24)	
Marital status									
Married/ cohabiting	77.7 (1921)	4.38 (4.19, 4.58)	<0.001	1.67 (1.60, 1.75)	0.11	23.72 (23.09, 24.37)	<0.001	13.67 (13.10, 14.26)	0.51
Single	22.3 (550)	3.78 (3.52, 4.05)		1.79 (1.66, 1.92)		19.55 (18.74, 20.40)		14.02 (13.12, 14.97)	
Financial hardship									
Yes	8.3 (204)	4.17 (3.73, 4.66)	0.90	1.82 (1.62, 2.04)	0.23	23.04 (21.51, 24.68)	0.47	13.40 (12.05, 14.89)	0.58
No	91.7 (2267)	4.20 (4.04, 4.38)		1.69 (1.62, 1.76)		22.43 (21.87, 23.00)		13.83 (13.30, 14.37)	

Notes: P-values and means (with 95% CLs) are from models with logarithmic diet variable. Means are back-transformed and report the weekly consumption rate of each food group. Analyses were adjusted for sex, age and occupational status prior to retirement and retirement year.

Supplementary figure 2. Changes in weekly **red meat** consumption frequency during retirement periods (-16 – +16 years from retirement) by sex (A), occupational status (B), marital status (C) and financial hardship (D). Models were adjusted for sex, age, and occupational status prior to retirement.

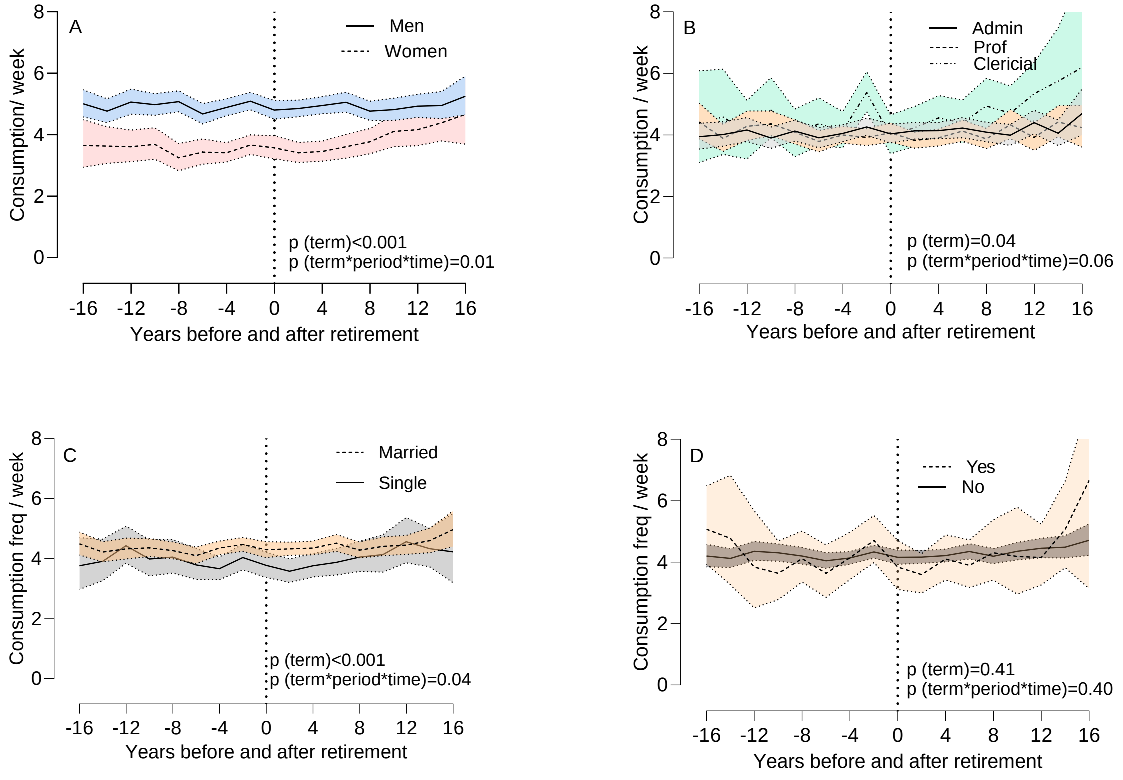

Supplementary figure 3. Changes in weekly **fish** consumption frequency during retirement periods (-16 – +16 years from retirement) by sex (A), occupational status (B), marital status (C) and financial hardship (D). Models were adjusted for sex, age, and occupational status prior to retirement. Term refers to the corresponding sociodemogsocio-demographic. UUDET

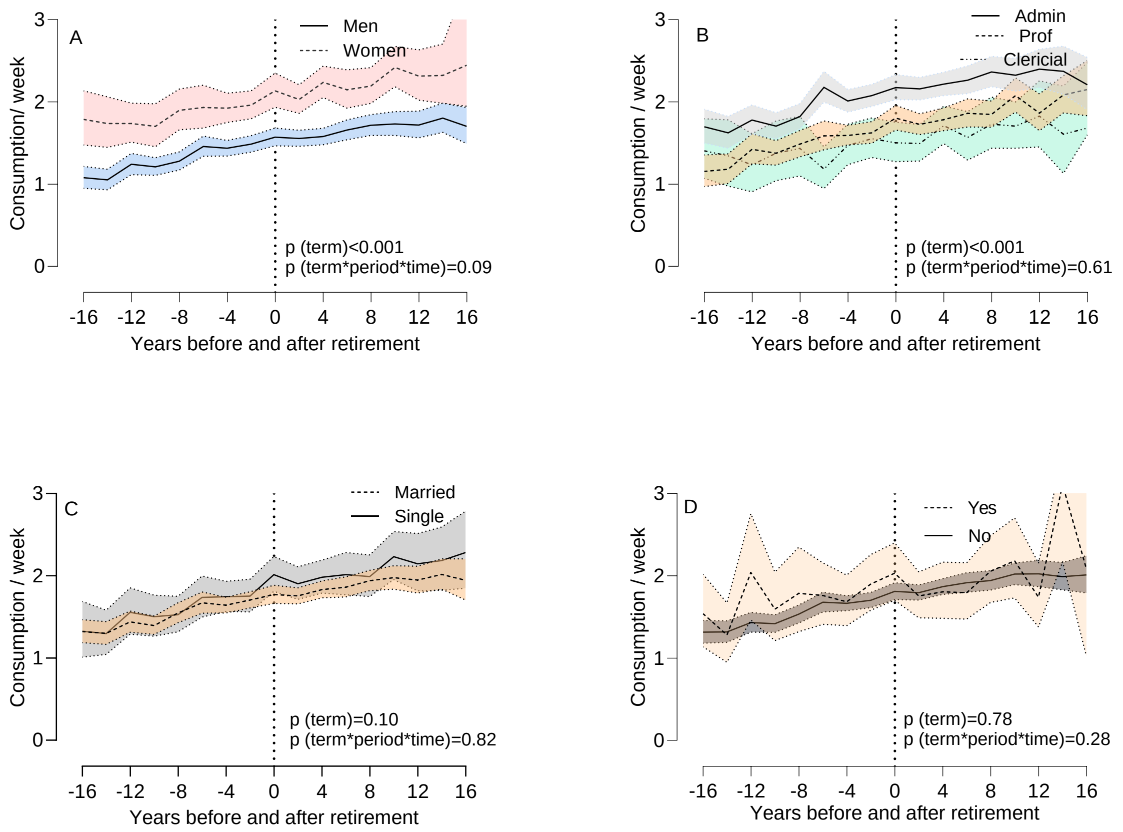

Supplementary figure 4. Changes in weekly **vegetables** consumption frequency retirement periods (-16 − +16 years from retirement) by sex (A), occupational status (B), marital status (C) and financial hardship (D). Models were adjusted for sex, age, and occupational status prior to retirement.

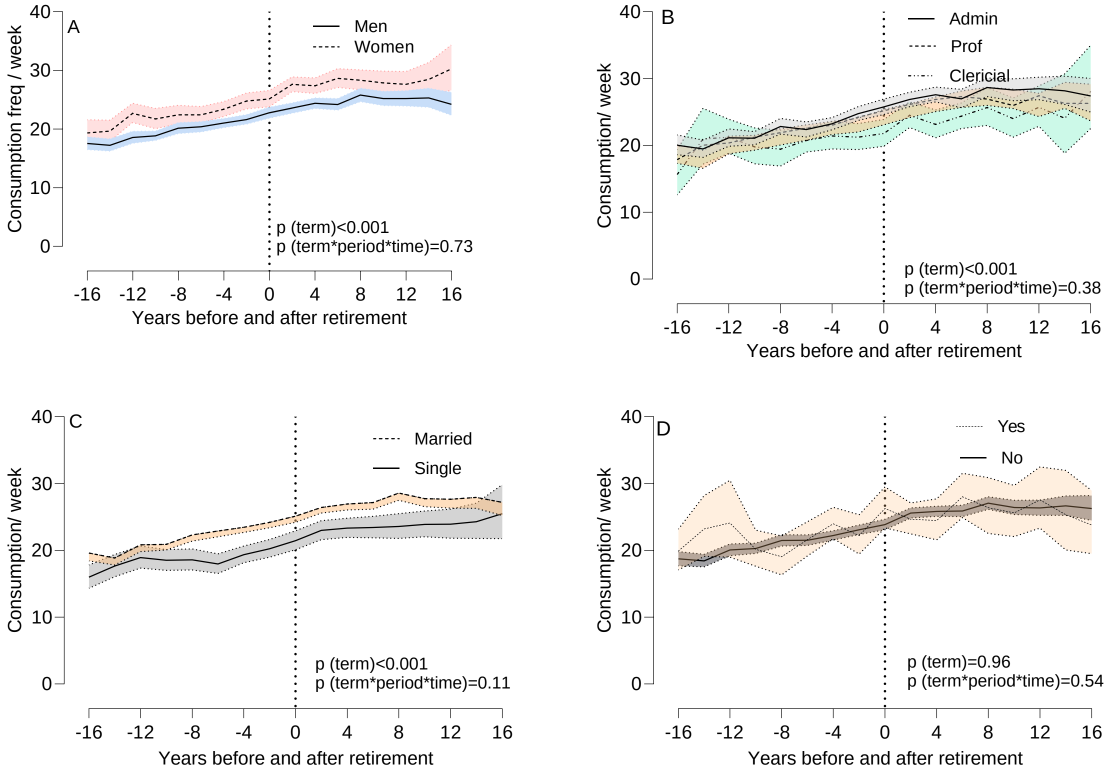

Supplementary figure 5. Changes in weekly **fruit** consumption frequency during retirement periods (-16 – +16 years from retirement)by sex (A), occupational status (B), marital status (C) and financial hardship (D). Models were adjusted for sex, age, and occupational status prior to retirement.

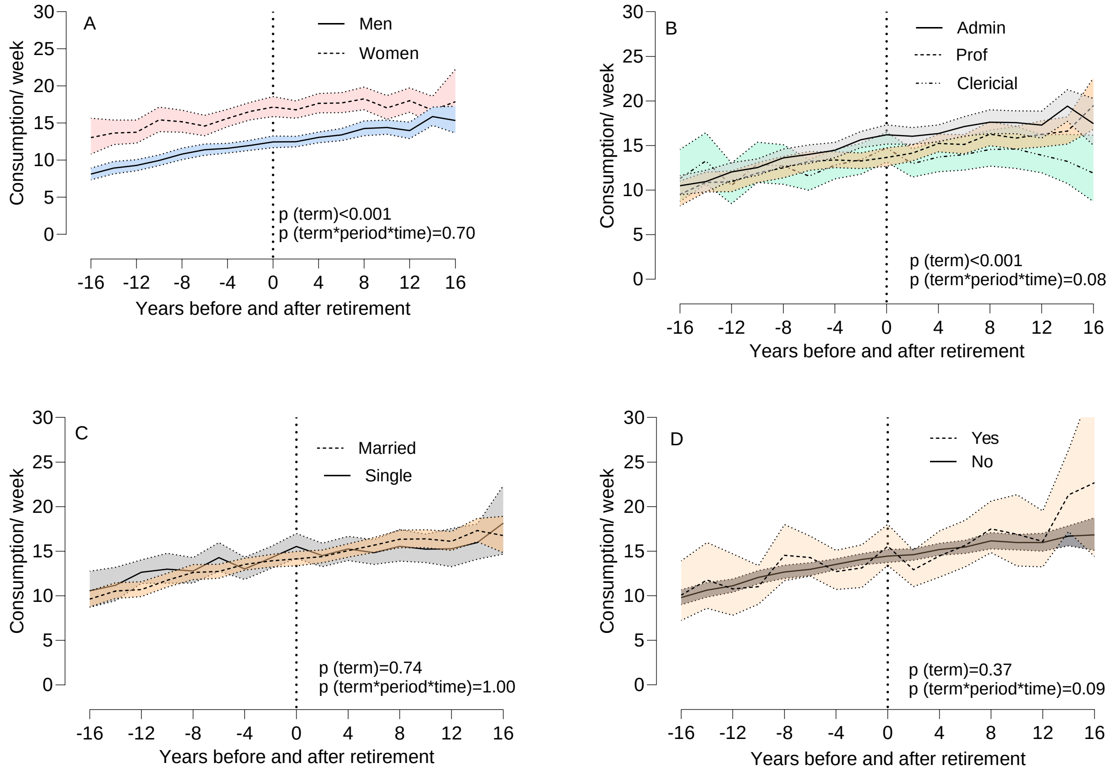

Supplement: online supplemental file 1 [file jech-79-2-s001.pdf]
